# An Account of Some Chemical Experiments on Tabasheer

**Published:** 1793

**Authors:** James Louis Macie


### XVII. *An Account of ſome chemical Experiments on Tabaſheer. By* James Louis Macie, *Eſq. F. R. S.*—Vide *Philoſophical Tranſactions of the Royal Society of London, Vol.* LXXXI. *for the Year* 1791. Part II. 4to. London, 1791.

THE Tabaſheer employed in theſe experiments conſiſted of ſeven different ſpecimens, one of which was Tabaſheer from Hydrabad *, the fineſt kind of this ſubſtance that can be purchaſed. This being in the greateſt quantity, and appearing to be the moſt homogeneous and pure, the experiments, we are told, were begun, and principally made, with it.

This Hydrabad Tabaſheer, in its general appearance, Mr. Macie obſerves, very much reſembled fragments of that variety of calcedony which is known to mineralogiſts by the name of *Cacholong*. Some pieces, he tells us, were quite opaque, and abſolutely white; while others poſſeſſed a ſmall degree of tranſparency, and had a bluiſh caſt. The latter, held before a lighted candle, appeared, it ſeems, very pellucid, and of a flame colour.

* See Vol. I. page 148.

 The

The pieces, he obſerves, were of various ſizes; the largeſt of them not exceeding two or three tenths of an inch cubic. Their ſhape was quite irregular; ſome of them bore impreſſions of the inner part of the bamboo againſt which they were formed.

This Tabaſheer, we are told, could not be broken by preſſure between the fingers; but by the teeth it was eaſily reduced to powder. On firſt chewing it felt gritty, but ſoon ground to impalpable particles.

Applied to the tongue, it adhered to it by capillary attraction.

It had a diſagreeable earthy taſte, ſomething like that of magneſia.

No light was produced either by cutting it with a knife, or by rubbing two pieces of it together, in the dark; but a bit of this ſubſtance, being laid on a hot iron, ſoon appeared ſurrounded with a feeble luminous *auréole*. By being made red hot, it was deprived of this property of ſhining when gently heated; but recovered it again, on being kept for two months.

Examined with the microſcope, it did not appear different from what it does to the naked eye.

Mr. Macie found that a quantity of this Tabasheer which weighed 75.7 gr. in air, weighed only 41.1 gr. in distilled water whose temperature was 52.5 F. which makes its specific gravity to be very nearly = 2.188.

Mr. Cavendish, he adds, who tried this same parcel when become again quite dry, found its specific gravity to be = 2.169.

In both the experiments to ascertain the specific gravity of this substance, great care, we are told, was taken that every bit was thoroughly penetrated with the water, and transparent to its very center, before its weight in the water was determined.

Mr. Macie gives an account of his experiments on this substance treated with water; with vegetable colours; at the fire; with acids; with liquid alkalies; with dry alkalies; and with other fluxes: after which follow his observations on the other specimens.

It appears from his experiments that five of the specimens consisted of genuine Tabasheer; but that those kinds, immediately taken from the plant, contained a certain portion of a vegetable matter, which was wanting in the specimens procured from the shops, and which our author thinks had probably been deprived of this admixture by calcination.

The nature of this ſubſtance, Mr. Macie obſerves, is very different from what might have been expected in the product of a vegetable. Its indeſtructibility by fire; its total reſiſtance to acids; its uniting by fuſion with alkalies in certain proportions into a white opaque maſs, in others into a tranſparent permanent glaſs; and its being again ſeparable from theſe compounds, entirely unchanged by acids, &c. (all of which circumſtances are proved by his experiments) ſeem, he thinks, to afford the ſtrongeſt reaſons to conſider it as perfectly identical with common *ſiliceous earth*.

Yet from pure quartz, he obſerves, it may be thought to differ in ſome material particulars; ſuch as in its fuſing with calcareous earth, in ſome of its effects with liquid alkalies, in its taſte, and its ſpecific gravity.

But its taſte, he thinks, may ariſe merely from its divided ſtate, for chalk and powdery magneſia both have taſtes, and taſtes which are very ſimilar to that of pure Tabaſheer; but when theſe earths are taken in the denſer ſtate of cryſtals, they are found to be quite inſipid; ſo Tabaſheer, he obſerves, when made more ſolid by expoſure to a pretty ſtrong heat, was no longer perceived, when chewed, to act upon the palate.

From Mr. Macie's experiments with liquid alkalies we learn that ſome liquid cauſtic vegetable alkali being heated in a phial, Tabaſheer was added to it, which diſſolved very readily and in conſiderable quantity; and that on expoſing this ſolution in a ſhallow glaſs to ſpontaneous evaporation in a warm room, at the end of a day or two it was converted into a firm, milky jelly, which in a few days more became whiter, and finally became quite dry and ſeparated from the glaſs. Theſe effects, however, with liquid alkali, he obſerves, are found on accurate compariſon not to be peculiar to this ſubſtance; for though it was found, on trial, that the powder of common flints, when boiled in liquid cauſtic alkali, was ſcarcely at all acted upon; and that the very little which was diſſolved was ſoon precipitated again, in the form of minute *flocculi*, on expoſing the ſolution to the air, and was immediately thrown down on the admixture of an acid; yet the precipitate obtained from *liquor ſilicum* by marine acid was diſcovered, it ſeems, even when dry, to diſſolve readily in this alkali, but while ſtill moiſt to do ſo very copiouſly, even without the aſſiſtance of heat; heat; and ſome of this ſolution, we are told, thus ſaturated with ſiliceous matter by ebullition, being expoſed to the air in a ſhallow glaſs, became a jelly by the next day, and the day after dried, and cracked, &c. exactly like the ſolution of Tabaſheer.

Mr. Macie obſerves that the aſhes, obtained by burning the bamboo, boiled in marine acid, left a very large quantity of a whitiſh inſoluble powder, which, fuſed at the blow-pipe with ſoda, effervefced, and formed a tranſparent glaſs. Only the middle part of the joints, we are told, was burned; the knots were ſawed off, leſt, being porous, Tabaſheer might be mechanically lodged in them. However, the great quantity of this remaining ſubſtance, he thinks, ſhows it to be an eſſential, conſtituent part of the wood.

The aſhes of common charcoal, digeſted in marine acid, left, it ſeems, in the ſame manner an inſoluble reſiduum which fuſed with ſoda with effervefcence, and formed glaſs; but the proportion of this matter to the aſhes, our author obſerves, was greatly leſs than in the foregoing caſe.

Mr. Macie gives the following account of a ſingular circumſtance, which, it ſeems, preſented itſelf after his experiments on this ſubſtance were were finiſhed.—" A green bamboo, cut in the " hot-houſe of Dr. Pitcairn, at Iſlington, was " judged to contain Tabaſheer in one of its " joints, from a rattling noiſe diſcoverable on " ſhaking it; but being ſplit by Sir Joſeph " Banks, it was found to contain, not ordinary " Tabaſheer, but a ſolid pebble, about the ſize " of half a pea.

" Externally this pebble was of an irregular " rounded form, of a dark-brown or black co- " lour. Internally it was reddiſh-brown, of " a cloſe dull texture, much like ſome martial " ſiliceous ſtones. In one corner there were " ſhining particles, which appeared to be cryſ- " tals, but too minute to be diſtinguiſhed even " with the microſcope.

" This ſubſtance was ſo hard as to cut glaſs!

" A fragment of it expoſed to the blow-pipe " on the charcoal did not grow white, contract " in ſize, melt, or undergo any change. Put " into borax it did not diſſolve, but loſt its " colour, and tinged the flux green. With " ſoda it efferveſced, and formed a round bead " of opaque black glaſs.

" Theſe two beads, digeſted in ſome perfectly " pure and white marine acid, only partially " diſſolved, and tinged this menſtruum of a

"greenish yellow colour; and from this solution Prussite of tartar, so pure as not, under many hours, to produce a blue colour with the above pure marine acid, instantly threw down a very copious Prussian blue."

